# The relationship between dietary patterns and blood mineral concentration among children in Hunan Province of China

**DOI:** 10.1186/s12889-023-16429-6

**Published:** 2023-08-10

**Authors:** Xiao-chen Yin, Wei-feng Wang, Zi-min Li, Yu-jie Duan, Ming Chen, Yuan-ni Wu, Yu-ming Hu

**Affiliations:** Department of Toxicology, Hunan Provincial Center for Disease Control and Prevention, Furong Road No. 450, Changsha, 410005 China

**Keywords:** Dietary patterns, Blood minerals, Correlation analysis

## Abstract

**Background:**

Minerals have crucial biological functions in metabolism and are primarily obtained through diet. As a result, various dietary patterns can impact blood mineral levels. The aim of this study was to investigate the correlation between dietary patterns and the concentration of calcium, magnesium, iron, zinc, and copper in the bloodstream.

**Methods:**

Three hundred eighty healthy children (53.7% male) were recruited in a region of Hunan Province in September 2019. We gathered basic information and measured physical proportions, along with completing a food frequency questionnaire (FFQ). Using principal component analysis (PCA), we determined dietary patterns. To analyze mineral levels in the blood, we used flame atomic absorption spectrometry (FAAS). We utilized linear regression models to investigate if certain dietary patterns are related to mineral concentration.

**Results:**

Three dietary patterns were identified: ‘Vegetables/Nuts,’ ‘Snacks/Beverages,’ and ‘Cereal/Beans.’ Children from high-income families (annual average income > 50,000 yuan) prefer the ‘Vegetables/Nuts’ dietary pattern (*P* = 0.004). In comparison, those from low-income families (annual average income < 20,000 yuan) prefer the ‘Snacks/Beverages’ dietary pattern (*P* = 0.03). Following adjustment for age, gender, guardian’s identity, education level, and annual household income. We found that an increase in the ‘Vegetables/Nuts’ pattern score (β = 0.153, CI: 0.053 ~ 0.253; *P* = 0.003) and ‘Snacks/Beverages’ pattern score (β = 0.103, CI: 0.002 ~ 0.204; *P* = 0.033) were significantly associated blood copper concentration.

**Conclusions:**

Household income was found to be associated with dietary behavior. Furthermore, higher blood copper concentration was significantly correlated with the ‘Vegetables/Nuts’ dietary pattern and ‘Snacks/Beverages’ dietary pattern, but the correlation is extremely low.

## Background

Essential elements like calcium (Ca), magnesium (Mg), iron (Fe), zinc (Zn), and copper (Cu) play crucial roles in the regulation and maintenance of various physiological functions in the human body despite their low requirements of them [1]. They are critical components of metalloenzymes and are involved in crucial biological processes, such as oxygen transport, free radical scavenging, and hormonal activity [2]. Ca is predominantly accumulated in bone tissue. Getting enough calcium as a child and teenager is crucial for strong bones [3]. Mg is abundant in human cells, plays an important role in 300 + metabolic reactions, and has been shown to be associated with Attention Deficit Hyperactivity Disorder (ADHD) [4–7]. Fe is a bivalent cation crucial for numerous physiological and cellular processes, and iron deficiency causes diverse health consequences [8]. Zn is the second most abundant metal in humans and is distributed unequally throughout different organs and tissues. In infants and early childhood, zinc supplementation can increase specific growth outcomes [9]. Cu is a crucial cofactor for more than twenty proteins [10]. Research shows that the level of copper in the serum is linked to obesity and various chronic illnesses in children [11, 12].

Dietary pattern analysis is a promising approach to understanding the complex relationship between diet and health [13]. An analysis based on nutrients alone may be confounded by the type of dietary pattern a person follows. They use a dietary pattern approach to overcome these limitations by considering how foods are eaten in combination. Poor diet in early life is a major modifiable risk factor with many health outcomes. Numerous studies have focused on the association between health outcomes and diet [14–16]. Research has revealed connections between the dietary patterns of children and adolescents with obesity [17–19]. Additionally, some studies have also found links between dietary patterns and cardiometabolic risk and depression [20, 21]. Studies in China examined the consumption of sugar-sweetened beverages and dairy by children and adolescents [22–24], but it did not specifically focus on dietary patterns. Recent studies conducted in China have revealed a strong correlation between a child's mineral status and lipid metabolism, and mineral status also has a significant impact on the symptoms of autism. [25, 26]. Meanwhile, Islam MR et al. proposed that alterations of serum mineral was associated with major depressive disorder [27]. Based on this information, we are considering the possibility that various dietary habits may indirectly impact the onset of specific illnesses by affecting the levels of essential minerals in the body.

The background level of minerals in children’s bodies is of great significance for monitoring the environment and evaluating children’s nutritional status [28–31]. While studies have shown a connection between mineral intake and various diseases [32–35], research on the correlation between dietary patterns and mineral levels is still lacking. Thus, this study aimed to investigate the correlation between the levels of calcium, magnesium, iron, zinc, and copper with different dietary patterns.

## Methods

### Study population

Participants were children enrolled in a cross-sectional study of blood minerals and eating behavior; this study is the first analysis of the recruitment visit, which occurred in Hunan Province, China, in September 2019. This study was approved by the Ethics Committee of Hunan Provincial Center for Disease Control and Prevention. Informed consent for the use of these children’s detection results and personal information in this study was obtained from their parents/legal guardians. Inclusion criteria were that the child was enrolled in local primary schools (either in the first or second year), aged 5 – 7 years, mentally competent, and had no serious medical problems affecting growth or appetite. All questionnaires were interviewer-administered.

Of the full sample of 392 children, 12 were excluded either due to missing or implausible dietary data.

### Dietary assessment and identification of dietary pattern

A validated 61-item food frequency questionnaire (FFQ) developed was employed to assess dietary intake. A common unit or portion size for each food was specified, and the participants' parents were asked to recall how often, on average, the child had consumed that item during the previous year. The selected frequency category for each food item was converted to a daily intake.

All data in the questionnaire were input using the software EpiData (version 3.1). According to the nutrient profile, the 61 items of the FFQ were allocated to 13 predefined food groups to identify eating patterns through Principal Component Analysis (PCA) (Table 2). The applicability of the PCA method was evaluated by the Kaiser–Meyer–Olkin coefficient (KMO) and Bartlett’s test of sphericity (BTS). In this study, the KMO value was 0.738, and the BTS test *P*-value was *P* < 0.0001, indicating the adequacy of the data for the factor analysis. The factors were rotated by an orthogonal transformation (varimax rotation function in SPSS software). Three dietary patterns were identified using multiple criteria: the diagram of eigenvalues, the scree plot, the interpretability of the factors, and the percentage of variance explained by the factors. The factor score for each pattern was calculated by summing intakes of all food groups weighted by their factor loadings. The food groups with the highest component loadings enabled interpretation of the pattern; groups with component loadings ≥|0.3| are shown in Table 2. To determine how closely each child’s diet aligned with the dietary pattern, scores were calculated by multiplying the component loadings of each food group by the child’s frequency of intake in that food group and the summing. Then scores were categorized into tertiles to differentiate children with lower scores (i.e., tertile 1, lower intake) from higher scores (i.e., tertile 3, higher intake). Finally, each score was converted to Z-scores with a mean of 0 and standard deviation of 1 to facilitate comparisons between 3 dietary patterns.

### Elemental analysis

After obtaining consent to participate in the study from each patient, 3 mL of blood was taken from the child to determine the concentration of the following minerals in blood: calcium, magnesium, iron, zinc, and copper. Before element detection, all the blood samples were stored at 0—4℃ for no more than 48 h.

Whole blood concentrations of calcium, iron, copper, magnesium, and zinc were determined by flame atomic absorption spectrometry (FAAS) instrument. Standard curves of these elements were conducted by serial dilution of the stock standard solutions before analyzing the metal on the instrument. Sample digestion was prepared by simply mixing 0.5 mL of blood sample and 4.5 mL of 0.5% Triton X-100 solution containing 1% HNO_3_ in a digestive tube. Then 0.5 mL sample dilution was added to 2 mL of 0.5% Triton X-100 dilution containing 1% HNO3 for mixing. They were detected immediately on the instrument after homogenizing.

### Covariates

Parents or other guardians provided relevant sociodemographic information, including the child’s age, gender, and the identity relationship between the actual guardian and the participants. The guardian relationship is divided into parents and others (others include grandparents, uncles/ aunts, brothers/ sisters). Meanwhile, guardians are required to provide their highest level of education attained and household income from all sources. Guardians' education level was classified into three categories: incomplete high school education, high school diploma, and completion of post-high school education. Similarly, participants' annual per capita household income was divided into three categories: less than 20,000 yuan, 20,000–50000 yuan, and more than 50,000 yuan. We used these categories to gain a better understanding of the group's demographics and to identify any potential correlations with other variables.

### Statistical analysis

All analyses were conducted using SPSS software (version 19.0 for Windows). The levels of calcium, magnesium, iron, zinc, and copper elements were analyzed using quantitative variables and assessed for normal distribution using the Kolmogorov–Smirnov test (K-S test). When representing normal distribution, we use mean ± standard deviation, while non-normal distribution is represented by median (IQR). Household income per capita was calculated as the total reported household income divided by the number of family members.

In bivariate analysis, the relationships between dietary patterns and potential confounders were initially explored by comparing the distributions of mean ± standard deviation (SD) of the potential confounders between the lowest and highest tertiles of dietary pattern categories (split at the tertile of dietary pattern scores). They were using dietary pattern scores as the continuous independent variable and minerals level as the continuous dependent variable, linear regression models were used to evaluate the primary study questions about diet and blood minerals. In multivariable models, the child’s gender, guardian’s educational level and income, and kinship to the child were added to each model. These variables were selected as confounders based on the results of the bivariate confounder analysis and prior research [36].

## Results

### General characteristics of study population

The sample comprised 176 (46.3%) girls and 204 (53.7%) boys. The mean age of the selection was 6.17 years (SD 0.68 years). Other descriptive statistics are shown in Table 1.

**Table 1:** Sociodemographic of a sample of 380 children and the guardian’s identity, education level, and household income per capita

Variable	Mean (SD) or %
**Age (years)**	6.17 (0.68)
**Gender**	
Male	53.7%
Female	46.3%
**Guardian**	
Parents	59.2%
Others^a^	40.8%
**Guardian’s education level**	
< High school	63.7%
High school diploma or GED^b^	27.3%
Some education beyond high school	9.0%
**Per-capita annual income (CNY)**	
< 20,000	24.5%
20,000 – 50,000	45.0%
> 50,000	30.5%

^a^Includes Grandparents, Uncles, Aunts, and brothers & sisters

^b^*GED* General Equivalency Diploma

### Dietary patterns

Using PCA and varimax rotation, three distinct dietary patterns were identified for the overall population, which explained 33.220% of the total variance in food intake. The factor loadings of the food groups contributing to the three dietary patterns are reported in Table 2 (groups with component loadings ≥|0.3| are bolded). Dietary pattern one, named ‘Vegetables/Nuts,’ explained 15.094% of the variance in food intake. High factor loadings on vegetables, homonemeae, nuts, aquatic foods, and fruits characterized this pattern. Pattern two, labeled ‘Snacks/Beverages,’ explained 9.725% of the variance in food intake and was characterized by high loadings on beverages, snacks, poultry, red meat, and nuts. Pattern three explained 8.401% of the variance in food intake and was named ‘Cereals/Beans.’ This pattern had high factor loadings on cereals, beans, fruits, and milk.

**Table 2:** Factor loadings of the 13 food groups in the four principal components extracted from the PCA of frequency of food intake data of 380 children

Food groups	Pattern 1	Pattern 2	Pattern 3
	**Vegetables/Nuts**	**Snacks/Beverages**	**Cereals/Beans**
Cereals	0.020	0.039	**0.711**
Beans	0.176	0.012	**0.647**
Red meat	**0.385**	**0.348**	-0.012
Poultry	**0.390**	**0.522**	-0.100
Eggs	0.049	-0.070	0.109
Aquatic foods	**0.515**	0.010	0.091
Milk	-0.052	0.217	**0.353**
Vegetables	**0.651**	-0.083	0.041
Homonemeae	**0.606**	-0.015	0.080
Fruits	**0.402**	0.182	**0.440**
Beverages	-0.079	**0.701**	0.114
Nuts	**0.633**	**0.324**	0.007
Snacks	0.041	**0.673**	0.187

### Concentrations of calcium, magnesium, iron, zinc, and copper in whole blood

The measurements of blood minerals for selected participants are summarized in Table 3. Based on the results of the K-W test, it appears that the concentration of the five minerals did not follow a normal distribution (*P* < 0.05). The median (IQR) for calcium, magnesium, iron, zinc, and copper were 69.63 (10.13), 41.09 (7.71), 434.70 (114.53), 4.55 (1.13), and 0.89 (0.19) mg/ L, respectively. The range of five minerals is consistent with previous studies [37, 38].

**Table 3:** 95% Confidence Interval of 5 Minerals

Minerals	Median (IQR) (mg/ L)	95% CI (mg/ L)
Calcium	69.63 (10.13)	55.55–89.72
Magnesium	41.09 (7.71)	32.93–54.46
Iron	434.70 (114.53)	310.28–648.73
Zinc	4.55 (1.13)	3.23–6.55
Copper	0.89 (0.19)	0.65–1.22

### Correlational analyses

Guardian’s income was associated with all three dietary patterns, such that children with higher scores on the Healthy pattern and lower scores on the Unhealthy pattern were more likely to have parents/legal guardians who have a higher income (Table 4) compared to children with lower scores on the corresponding pattern.

**Table 4:** Cross-sectional associations between sociodemographic characteristics and dietary patterns among 380 children enrolled in a study on blood trace elements concentration and eating behavior

		**‘Vegetables/Nuts’**		**‘Snacks/ Beverages’**		**‘Cereals/ Beans’**	
**Sociodemographic variables**	**n**	**Highest scores**	**Lowest scores**	**Highest scores**	**Lowest scores**	**Highest scores**	**Lowest scores**
**Gender**							
Male	204	70	64	70	71	74	59
Female	176	57	62	57	55	53	67
*P* value		0.491		0.844		0.068	
**Guardian**							
Parents	225	77	64	78	75	75	79
Others^a^	155	50	62	49	51	52	47
*P* value		0.115		0.758		0.553	
**Guardian education level**							
Incomplete high school education	242	83	79	84	76	81	79
High school diploma or GED^b^	104	32	36	32	37	35	37
Some education beyond high school	34	12	11	11	13	11	10
*P* value		0.830		0.630		0.940	
**Household income per capita (CNY)**							
< 20,000	93	21	37	41	19	21	37
20,000–50000	171	54	60	51	71	65	49
> 50,000	116	52	29	34	37	40	41
*P* value		0.004**		0.003**		0.036*	

^a^Includes Grandparents, Uncles/ Aunts, and brothers/ sisters

^b^*GED* General Equivalency Diploma

^**^ *P* < 0.01, * *P* < 0.05

In regression analysis (Table 5), blood copper concentration was related to a 0.14 higher Healthy dietary pattern SD after adjustment for potential confounders (95% Confidence Interval (CI) 0.038 to 0.242, *P* = 0.004), and copper concentration was also associated with a 0.103 SD higher Unhealthy dietary pattern score (95% CI 0.002 to 0.204, *P* = 0.045), and neither dietary pattern resulted in a copper concentration exceeding/below the normal range. Concentrations of calcium, iron, magnesium, and zinc were not associated with dietary pattern scores.

**Table 5:** Cross-sectional associations between trace elements concentration and dietary patterns among 380 children enrolled in a study on mineral status and eating behavior

Dietary Pattern	Trace Elements	Unadjusted β (95% CI) ^a^	Adjusted β (95% CI) ^b^
Vegetables/Nuts	Ca	-0.051 (-0.152, 0.050)	-0.047 (-0.149, 0.056)
	Fe	0.052 (-0.049, 0.153)	0.050 (-0.053, 0.152)
	Cu	0.153 (0.053, 0.253) **	0.140 (0.038, 0.242) **
	Mg	0.051 (-0.050, 0.152)	0.056 (-0.047, 0.159)
	Zn	0.060 (-0.041, 0.161)	0.062 (-0.041, 0.166)
Snacks/ Beverages	Ca	0.042 (-0.059, 0.143)	0.045 (-0.057, 0.146)
	Fe	-0.004 (-0.105, 0.098)	-0.001 (-0.103, 0.100)
	Cu	0.109 (0.009, 0.210) *	0.103 (0.002, 0.204) *
	Mg	0.061 (-0.039, 0.162)	0.061 (-0.041, 0.163)
	Zn	-0.033 (-0.134, 0.068)	-0.030 (-0.132, 0.072)
Cereals/ Beans	Ca	-0.071 (-0.172, 0.030)	-0.067 (-0.170, 0.037)
	Fe	-0.057 (-0.158, 0.044)	-0.063 (0.167, 0.040)
	Cu	0.019 (-0.082, 0.120)	-0.001 (-0.105, 0.103)
	Mg	-0.006 (-0.108, 0.095)	-0.004 (-0.108, 0.101)
	Zn	-0.092 (-0.192, 0.009)	-0.094 (-0.197, 0.010)

^a^All estimates are from linear regression models with the dietary pattern as the independent variable and elements concentration as the dependent variable

^b^Adjusted for child sex, guardian’s education level and income, and their kinship to the child

^**^P < 0.01; *P < 0.05

## Discussion

This is the first study to explore the association between dietary patterns and blood minerals level. In the past 20 years, the nutritional status of children in China has greatly improved, but there are still unhealthy diets and insufficient intake of micronutrients [39]. Dietary patterns directly affect children’s nutritional and health status. Therefore, studying the current situation of dietary patterns and evaluating the association between dietary patterns and micronutrients will provide a practical solution to improve the nutritional intake of children.

This study aimed to identify dietary patterns within school-age children and to explore the correlation with blood minerals (calcium, iron, copper, magnesium, and zinc) concentration. In this sample of school-age children, three dietary patterns were identified – ‘Vegetables/Nuts’ dietary pattern, ‘Snacks/Beverages’ dietary pattern, ‘Cereals/Beans’ dietary pattern. One of the primary findings was that household income was associated with differences in pattern behavior. Children with higher family incomes tend to the ‘Healthy-conscious’ dietary pattern, and middle-income families tend to prefer the traditional ‘Cereals/Beans’ dietary pattern.

Income and food cost are the two most important determinants of dietary convergence in developing nations [40]. While globalization presents a chance for a greater intake of healthy and diverse foods in economies in transition, it also permits an increase in the consumption of inexpensive, energy-dense foods [41]. Several studies described the positive association between household income and ‘Healthy’ dietary patterns [42, 43]. And cross-sectional studies found that the ‘Unhealthy’ dietary pattern was inversely associated with income [44, 45]. The inverse relationship could be due to the high cost of healthy diets. [46–48]. A study based on the China Health and Nutrition Survey revealed that an increase in family income boosts protein and fat intake but has a negative correlation with carbohydrate consumption [49]. And individuals with higher salaries tend to consume a wider variety of foods. As expected, a significant positive correlation exists between income and dietary knowledge, as higher-income persons are more health-conscious and have greater access to health information [50–52].

Another finding was that blood copper concentration was positively correlated with ‘Healthy-conscious’ dietary pattern scores and ‘Snacks/Beverages’ dietary pattern scores after accounting for confounders, although the regression coefficient is low (*P* < 0.05, β < 0.3). Copper metabolism is regulated by physiologic demand, but the mechanisms involved have not been elucidated. And copper deficiency does not occur frequently, and it most often occurs in patients with Menkes disease (MD), a genetic disorder of impaired copper homeostasis. Excess copper has also been reported in humans, most often being associated with another rare genetic disorder, Wilson’s disease (WD).

It is well known that none of the five minerals can be synthesized in the human body and can only be consumed through foods. The best dietary sources of copper are shellfish, seeds, nuts, organ meats, and bran cereal. High-loading nuts and homonemeae in the ‘Healthy-conscious’ pattern are rich in copper (> 2.4 μg/g). In addition, about 55%—75% of dietary copper is absorbed, which is considerably higher than other minerals [53]. Therefore, we speculate that elevated blood copper levels are a short-term effect caused by high copper dietary intake. Moreover, mRNA levels for many proteins involved in copper homeostasis in mammals (e.g., CTR1, ATP7A, and ATP7B) do not change in response to dietary copper intake levels, demonstrating a lack of control at the level of gene transcription or transcript stability. Regulation of copper intake and efflux may instead be controlled at a posttranscriptional level, predominantly by protein trafficking, as exemplified by the copper-transporting ATPases moving from the TGN to either the enterocyte BLM (APT7A) or the canalicular membrane of hepatocytes (ATP7B) when copper is in excess [54]. Copper metabolism is best known to be influenced by iron. It has been suggested that iron can interfere with copper utilization, and high iron consumption can interfere with copper absorption in infants and adults [55]. Plant components in vegetables and tea (e.g., polyphenols, phytates) and soft drinks inhibit iron absorption, which may also contribute to an indirect increase in blood copper levels [56].

Therefore, we propose that dietary patterns cannot reflect children’s long-term mineral needs. Firstly, the proportion of mineral elements in the blood is extremely low, making the blood concentration highly susceptible to a short-term diet. Meanwhile, the interaction of mineral elements also makes their concentration unpredictable. In August 2021, the National Health Commission of China issued a notice that trace elements testing of children shall not be carried out as a medical examination item unless the diagnosis and treatment needs. The blood concentration of trace elements in healthy individuals remains relatively stable because they are strictly regulated [57–64]. Evaluating the trace elements status in healthy children through blood concentration still needs further study.

There are several limitations to our study. Firstly, the research may be limited in its ability to generalize conclusions due to a small sample size and narrow age range among participants. Secondly, the research was carried out only in one specific city, which may restrict the generalization of the results to other areas. Thirdly, the study participants were healthy individuals, but low blood levels due to element deficiency are usually accompanied by obvious clinical symptoms. Finally, we cannot exclude the possibility of residual confounding in the analysis due to unmeasured or imprecisely measured factors. It is possible, for example, that dietary intake is only one component of an overall lifestyle that affect the content of blood minerals. Such passive smoking [65, 66] and high-intensity exercise [67, 68] can lead to significant changes in blood minerals.

Mineral homeostasis is a complex and highly regulated process involving acquisition, utilization, storage, and efflux. Although some limitations may apply, blood mineral concentration is still used as the standard for evaluating trace elements status in patients. To comprehensively evaluate minerals status, both laboratory tests and the clinical assessment of trace elements deficit symptoms might be required.

## Conclusions

This study demonstrated that dietary patterns had no effect on blood mineral levels and found an association between household income and dietary patterns. Adequate minerals are essential for children’s growth and development. Therefore, the food intake pattern of children should receive greater attention from public health policies, and the complex relationship between dietary patterns and mineral still needs further study.

## Data Availability

All data generated or analyzed during this study are included in this published article.
